# *t*/*k*-Diagnosability of Regular Networks under the Comparison Model

**DOI:** 10.3390/s24072303

**Published:** 2024-04-04

**Authors:** Jiarong Liang, Mengdie Xiao, Zhi Lin, Changzhen Li

**Affiliations:** 1 School of Computer, Electronics and Information, Guangxi University, Nanning 530004, China; 13977106752@163.com (J.L.); 2113301049@st.gxu.edu.cn (M.X.); 2113301024@st.gxu.edu.cn (Z.L.); 2 School of Public Policy and Management, Guangxi University, Nanning 530004, China

**Keywords:** *t*/*k*-diagnosability, *t*/*k*-diagnosis algorithm, interconnection networks, comparison model

## Abstract

As multiprocessor systems continue to grow in processor scale, the incidence of faults also increases. As a result, fault diagnosis is becoming a key mechanism for maintaining the normal operation of multiprocessor systems. To explore more effective diagnostic methods, Somani et al. introduced a generalized pessimistic diagnostic strategy, named t/k-diagnosis, in which all faulty nodes are isolated in a set of nodes and at most k fault-free nodes are misdiagnosed, provided that the quantity of faults is limited by t. By imposing certain conditions or restrictions, the t/k-diagnosability of some regular networks under the Preparata, Metze, and Chien (PMC) model has been determined. However, the t/k-diagnosability of many networks under the comparison model remains unidentified. In this paper, we provide new insights into the study of t/k-diagnosability under the comparison model. After introducing some new notions, such as the 0-test unit, 0-test set and 0-test subgraph, under the comparison model, we study the relationship in a system *G* between the 0-test subgraphs and the components of G−F, where *F* is the set of faulty nodes, and we obtain some important correlation properties. Based on these results, we study t/k-diagnosability under the comparison model. As a result, the t/k-diagnosability of some regular interconnection networks can be efficiently determined.

## 1. Introduction

With the rapid advancement of information technology, the precision of very large-scale integration (VLSI) is becoming increasingly sophisticated. Today’s supercomputers may have thousands of processors. Take the US supercomputer Summit, which was crowned the world’s fastest super computer in 2018 and 2019, for example; it has 9216 processors. The large scale of its processor numbers may cause many unreliability problems. Therefore, reliability is an important issue to consider in the design, operation, and maintenance of such a large-scale multiprocessor system. To maintain system reliability, it is necessary to quickly identify all faults. The procedure of recognizing faults is known as fault diagnosis. System-level diagnosis is considered an ideal fault diagnostic method [1].

Many important diagnostic strategies have been proposed in the course of the development of system-level fault diagnosis theory. Among them, the diagnostic capability of the original diagnostic strategy introduced by Preparata et al. [1], named t-diagnosis, is relatively weak. To improve the diagnostic capability, another important diagnostic strategy, called t/k-diagnosis [2], which requires all faulty nodes to be isolated in a set of nodes, was proposed by Somani et al. In this approach, at most *k* nodes can be misdiagnosed if the fault node number does not exceed *t*. For the system *G*, its t/k-diagnosability is the maximum value of *t* satisfying the condition that *G* is t/k-diagnosable. For example, the hypercube is an important network topology, which has been applied to many parallel and distributed systems such as iWarp [3] and Cray T3D [4]. For n⩾4 and n⩾k⩾1, it is proved by Somani et al. [2] that the hypercube $Q_{n}$ is $[\left(k+1\right)n-\frac{\left(k+1)(k+2\right)}{2}+1]/k$-diagnosable. The t/k-diagnosability of several networks under the PMC model has been determined, including hypercubes [2], star graphs [2,5], mesh-based systems [2], and bijective connection (BC) networks [6,7]. Recently, by utilizing the properties of the 0-test subgraph under the PMC model, Lin et al. [8] studied the t/k-diagnosability of regular graphs under the PMC model.

It is well known that there are three system-level diagnosis models: the BGM model [9], the comparison model [10], and the PMC model. The BGM model is not often used in the existing literature as a fault diagnosis model due to its flaws. It is worth mentioning as Sengupta and Dahbura state[10], the comparison diagnosis model can be obtained by generalizing the PMC model. In other words, in terms of diagnosis model, the comparison model is often more suitable than the PMC model for studying the system fault diagnosis. However, to the best of our knowledge, to date, few studies have investigated t/k-diagnosability under the comparison model. In this paper, we study the problem of t/k-diagnosability for regular networks under the comparison model.

The main contributions of this paper are described below.

- To study t/k-diagnosability based on the comparison model, the paper introduces some important definitions, such as the 0-test unit and 0-test subgraph, and present their related properties;
- We present the description of the t/k-diagnosability of regular networks under the comparison model. At the same time, we propose a t/k-diagnosis algorithm for regular networks under the comparison model, which is, to the best of our knowledge, the first such t/k-diagnosis algorithm for regular networks under the comparison model;
- We give the t/k-diagnosabilities for some famous network systems such as hypercube networks, star networks, complete cubic networks, and so on.

The rest of the paper is organized as follows. In the following section, some necessary terminologies and notations are presented. We introduce the definition and properties of the 0-test subgraph in Section 3. Section 4 presents the main results of this paper. We discuss some applications in Section 5. Section 6 concludes the paper.

## 2. Preliminaries

A multiprocessor system can be modeled as a graph G(V,E), with V(G) being the node set and E(G) being the edge set. For x∈V(G), N(x) is the set of all the neighbors of *x*, and $\deg_{G}\left(x\right)$ is the degree of *x* in *G*. Let $\Delta \left(G\right)=\max_{x\in V(G)}\;\deg\left(x\right)$ and $\delta \left(G\right)=\min_{x\in V(G)}\;\deg\left(x\right)$. Then, $N\left(A\right)=\underset{x\in A}{⋃}N(x)-A$ and $N_{B}\left(A\right)=N\left(A\right)\cap B$, where A⊂V(G) and B⊂V(G).

**Figure 1:**
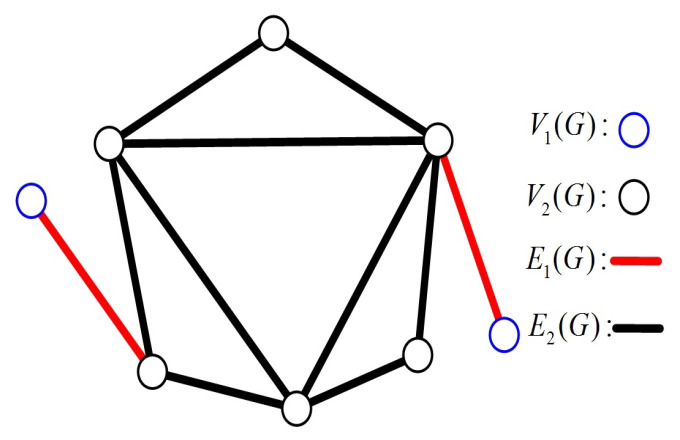
Illustration of pendant nodes and pendant edges.

In a connected graph, nodes with degree 1 are known as pendant nodes. A pendant edge is incident to at least one pendant node. Then, all the nodes and edges in *G* can be classified into two types: pendant and non-pendant. Let $V_{1}\left(G\right)$ and $V_{2}\left(G\right)$ be the sets of pendant nodes and non-pendant nodes, and let $E_{1}\left(G\right)$ and $E_{2}\left(G\right)$ be the sets of pendant edges and non-pendant edges, respectively, (see Figure 1). Then, we have the following properties.

**Lemma** **1.** *If G is a connected graph with |V(G)|≥3, then $|E_{1}\left(G\right)|=|V_{1}\left(G\right)|$.*

**Proof** **of** **Lemma** **1.** Since *G* is connected with |V(G)|≥3, there exist no edges whose two endpoints are pendant nodes. That is, each pendant edge corresponds to a different pendant node (see Figure 1). Therefore, $|E_{1}\left(G\right)|=|V_{1}\left(G\right)|$.    □

**Lemma** **2.** *$G-V_{1}\left(G\right)$ is connected.*

**Figure 2:**
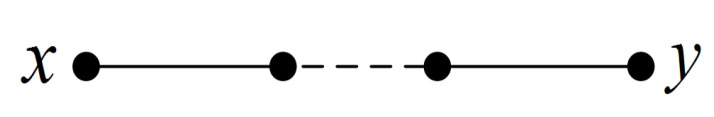
An illustration of Lemma 2.

**Proof** **of** **Lemma** **2.** For any two nodes $x,y\in V_{2}\left(G\right)$, since *G* is connected, there exists a path that connects *x* and *y* (see Figure 2). Since each node in $V_{1}\left(G\right)$ has degree 1, the path will not pass through any node in $V_{1}\left(G\right)$. Thus, there is a path that connects *x* and *y* in $G-V_{1}\left(G\right)$. Therefore, $G-V_{1}\left(G\right)$ is connected.    □

**Lemma** **3.** *Let G=(V,E) be a connected graph satisfying |V|≥3 and Δ(G)≥2. Then, $|V_{2}\left(G\right)|\geq \frac{|V(G)|-2}{\Delta (G)-1}$.*

**Proof** **of** **Lemma** **3.** We have $V_{1}\left(G\right)=V\left(G\right)-V_{2}\left(G\right)$. By Lemma 2, $G-V_{1}\left(G\right)$ is connected. That is, all nodes in $V_{2}\left(G\right)$ are connected by edges in $E_{2}$. Clearly, $|E_{2}\left|\geq \right|V_{2}|-1$. Assume the average degree in $V_{2}$ is *a* with 2≤a≤Δ(G). By Lemma 1, $|E_{1}\left(G\right)|=|V_{1}\left(G\right)|$. According to Euler’s handshaking lemma, we have

$$\begin{array}{c} |V_{1}\left(G\right)|+a|V_{2}\left(G\right)|=2(|E_{1}\left(G\right)|+|E_{2}\left(G\right)\left|\right)\\ \Rightarrow |V_{1}\left(G\right)|+a|V_{2}\left(G\right)|\geq 2(|V_{1}\left(G\right)|+|V_{2}\left(G\right)\left|-1\right)\\ \Rightarrow \left(a-2\right)|V_{2}\left(G\right)|\geq |V_{1}\left(G\right)|-2\\ \Rightarrow \left(a-2\right)|V_{2}\left(G\right)|\geq |V\left(G\right)|-|V_{2}\left(G\right)|-2\\ \Rightarrow |V_{2}\left(G\right)|\geq \frac{|V(G)|-2}{a-1}\geq \frac{|V(G)|-2}{\Delta (G)-1}. \end{array}$$

Therefore, $|V_{2}\left(G\right)|\geq \frac{|V(G)|-2}{\Delta (G)-1}$.    □

In system-level diagnosis, the PMC model [1] and comparison model [10] are two widely adopted diagnostic models. Under the comparison model, a comparator will distribute a task to its two adjacent nodes and compare the responses they provide. The comparison of nodes *x* and *y* performed by *z* is denoted by ${\left(x,y\right)}_{z}$, where (z,x) and (z,y) denote two test edges, respectively. The outcome of test ${\left(x,y\right)}_{z}$ is represented by $\sigma {\left(x,y\right)}_{z}$. In Table 1, the invalidation rules for the comparison model are summarized. By Table 1, if $\sigma {\left(x,y\right)}_{z}=0$, all three nodes are fault-free or the tester *z* is faulty. Moreover, if *x* and *z* are fault-free, we can identify *y* as fault-free by $\sigma {\left(x,y\right)}_{z}=0$ or as faulty by $\sigma {\left(x,y\right)}_{z}=1$.

A collection of all the test results is called a syndrome σ. For a given syndrome σ, *F* is called an allowable faulty set if σ can be produced from *F*, i.e., if the following two conditions hold:

(a) $\sigma {\left(x,y\right)}_{z}=0$ for x,y,z∈V−F;
(b) $\sigma {\left(x,y\right)}_{z}=1$ for z∈V−F and x∈F (or y∈F).

For a given syndrome σ, if there are several allowable faulty sets $F_{1},F_{2},\ldots ,F_{r}$, we cannot accurately diagnose the set. As a result, the faulty nodes can only be isolated into a set *F*, such that $F=F_{1}\cup F_{2}\cup \ldots \cup F_{r}$.

## 3. 0-Test Subgraph under the Comparison Model

**Figure 3:**
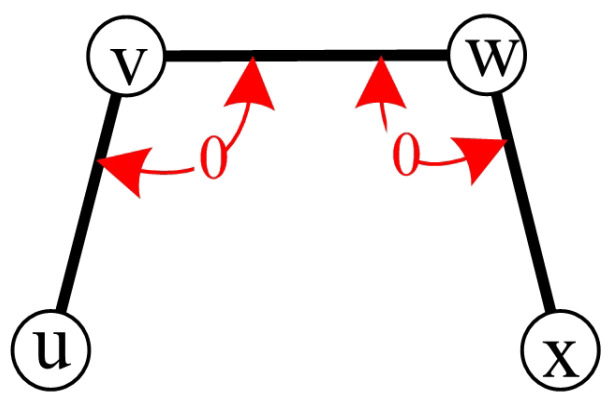
Illustration of a 0-test set.

For a given a syndrome σ under the comparison model, test ${\left(u,w\right)}_{v}$ is a 0-test unit if $\sigma {\left(u,w\right)}_{v}=0$, where (u,v) and (w,v) are two test edges. The two tests, ${\left(u,w\right)}_{v}$ and ${\left(v,x\right)}_{w}$, belong to the same 0-test set because they share at least one common test edge (see Figure 3). The graph induced by a 0-test set is called a 0-test subgraph.

**Figure 4:**
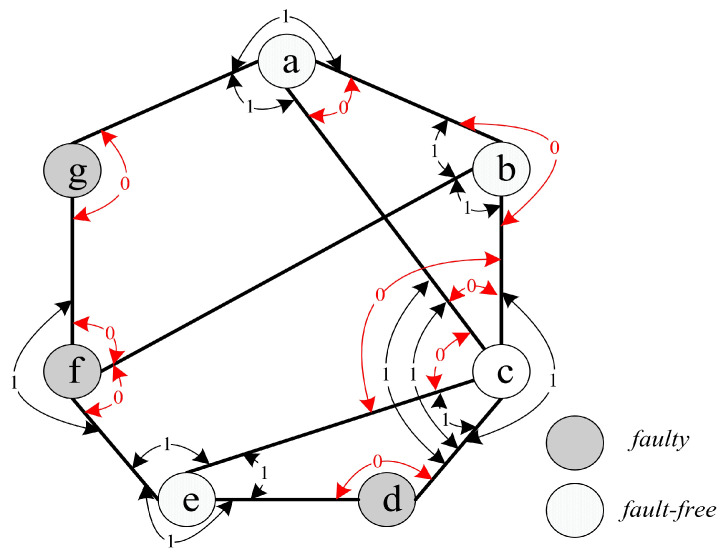
Syndrome σ of graph *G* with 7 nodes a,b,c,d,e,f,g.

**Figure 5:**
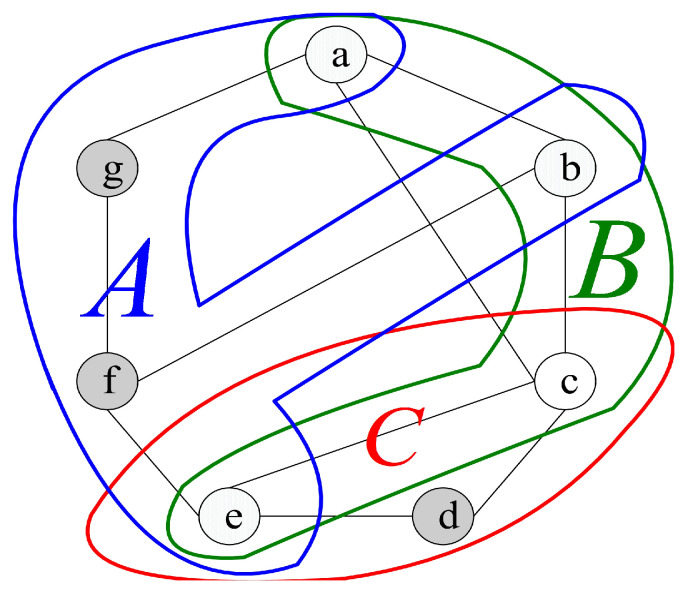
0-test sets A,B and *C* of graph *G* with 7 nodes a,b,c,d,e,f,g.

**Figure 6:**
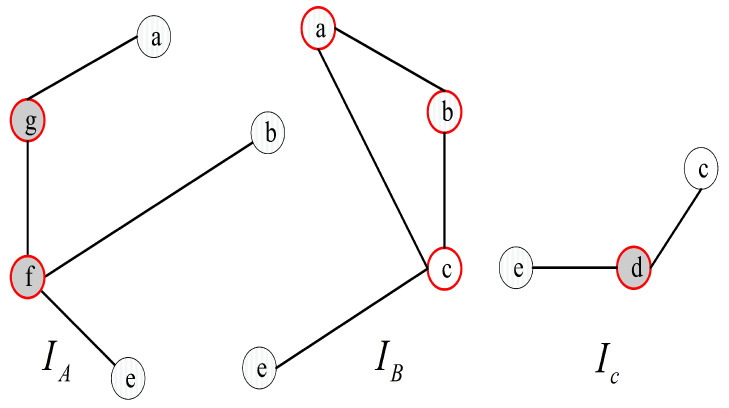
$T_{0}\left(G\right)$ of graph *G* with 7 nodes a,b,c,d,e,f,g.

For instance, we have V(G)={a,b,c,d,e,f,g} (see Figure 4). The syndrome under the comparison model is represented in Figure 4, where $\sigma {\left(b,c\right)}_{a}=0$, $\sigma {\left(a,c\right)}_{b}=0$, $\sigma {\left(a,b\right)}_{c}=0$, $\sigma {\left(b,e\right)}_{c}=0$, $\sigma {\left(a,e\right)}_{c}=0$, $\sigma {\left(c,e\right)}_{d}=0$, $\sigma {\left(g,b\right)}_{f}=0$, $\sigma {\left(b,e\right)}_{f}=0$, and $\sigma {\left(f,a\right)}_{g}=0$, and the outcomes of other tests are 1. Hence, there are nine 0-test units, ${\left(b,c\right)}_{a}$, ${\left(a,c\right)}_{b}$, ${\left(a,b\right)}_{c}$, ${\left(b,e\right)}_{c}$, ${\left(a,e\right)}_{c}$, ${\left(c,e\right)}_{d}$, ${\left(g,b\right)}_{f}$, ${\left(b,e\right)}_{f}$, and ${\left(f,a\right)}_{g}$. Furthermore, there are three 0-test sets, *A*={${\left(f,a\right)}_{g}$, ${\left(g,b\right)}_{f}$, ${\left(b,e\right)}_{f}$}, *B*={${\left(b,c\right)}_{a}$, ${\left(a,c\right)}_{b}$, ${\left(a,b\right)}_{c}$, ${\left(b,e\right)}_{c}$, ${\left(a,e\right)}_{c}$}, and *C*={${\left(c,e\right)}_{d}$} (see Figure 5). Then, let $I_{A}$, $I_{B}$, and $I_{C}$ be the 0-test subgraphs induced by 0-test sets *A*, *B*, and *C*, respectively, (see Figure 6). The set of all 0-test subgraphs of *G* is written as $T_{0}\left(G\right)=\left\{I_{A},I_{B},I_{C}\right\}$. For any $X\in T_{0}\left(G\right)$, |V(X)|≥3.

Let *H* be a 0-test set under the comparison model. Let τ(H) represent the set consisting all testers in *H*. Clearly, all the testers in *H* are connected in *H* or |τ(H)|=1. In the previous example, we have τ(A)={g,f}, τ(B)={a,b,c}, and τ(C)={d}. Then, we have the following properties.

**Lemma** **4.** *Let H be a 0-test set of G under the comparison model. Either all the nodes in H are fault-free or each node in τ(H) is faulty.*

**Proof** **of** **Lemma** **4.** For arbitrary ${\left(b,c\right)}_{a}\in H$, $\sigma {\left(b,c\right)}_{a}=0$. By Table 1, all the nodes of *a*, *b*, and *c* are fault-free or tester *a* is faulty. Let ${\left(a,e\right)}_{c}$ be another 0-test unit in *H* that has a common test edge with ${\left(b,c\right)}_{a}$. We have $\sigma {\left(a,e\right)}_{c}=0$. If *a*, *b*, and *c* are fault-free, *e* is also fault-free because $\sigma {\left(a,e\right)}_{c}=0$. This process continues until all 0-test units in *H* have been examined. Therefore, all the nodes in *H* are fault-free. Otherwise, *a* is faulty, since $\sigma {\left(a,e\right)}_{c}=0$, *c* is also faulty by Table 1. As a result, all the nodes in τ(H) are faulty.    □

**Lemma** **5.** *Assume that F represents a fault set of G. For any component C of G−F, C is a 0-test subgraph under the comparison model.*

**Figure 7:**
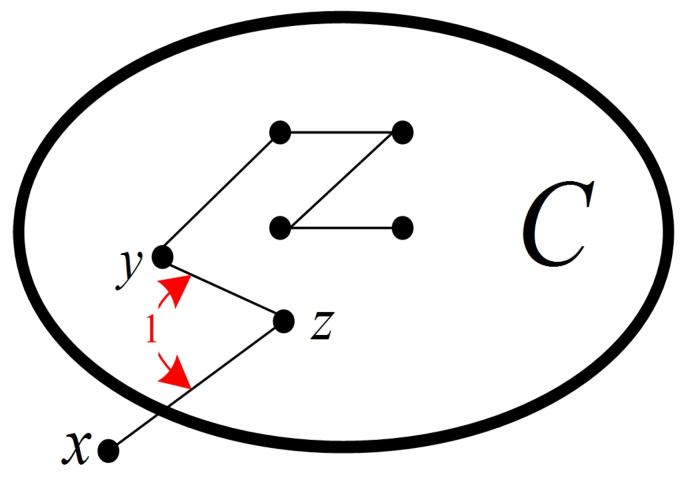
An illustration of Lemma 5.

**Proof** **of** **Lemma** **5.** Since *C* is a component of G−F, *C* is connected, and all the nodes in *C* are fault-free with N(C)⊆F. Hence, under the comparison model, any test in *C* is a 0-test unit. Therefore, *C* belongs to a 0-test subgraph $S\in T_{0}\left(G\right)$. For any x∈N(C), without loss of generality, suppose that (x,z),(y,z)∈E(G) with y,z∈V(C) (see Figure 7). Then, we have $\sigma {\left(x,y\right)}_{z}=1$. Hence, x∉V(S). That is, each node in N(C) does not belong to *S*. Therefore, C=S.    □

**Lemma** **6.** *Let S represent a 0-test subgrapht corresponding a component C of G−F under the comparison model. Then, $\tau \left(S\right)=V_{2}\left(C\right)$.*

**Proof** **of** **Lemma** **6.** Since *C* is connected and all the nodes in *C* are fault-free, we have $V_{2}\left(C\right)\subseteq \tau \left(S\right)$. For an arbitrary 0-test unit ${\left(x,y\right)}_{a}$ in *S*, tester *a* has at least two neighbors *x* and *y* in *C*. Thus, $a\in V_{2}\left(C\right)$. Then, we have $V_{2}\left(C\right)⊇\tau \left(S\right)$. Therefore, $V_{2}\left(C\right)=\tau \left(S\right)$.    □

**Lemma** **7.** *Let F be a fault set of G and let $S\in T_{0}\left(G\right)$ with |τ(S)|>|F|; then, S is a component of G−F.*

**Figure 8:**
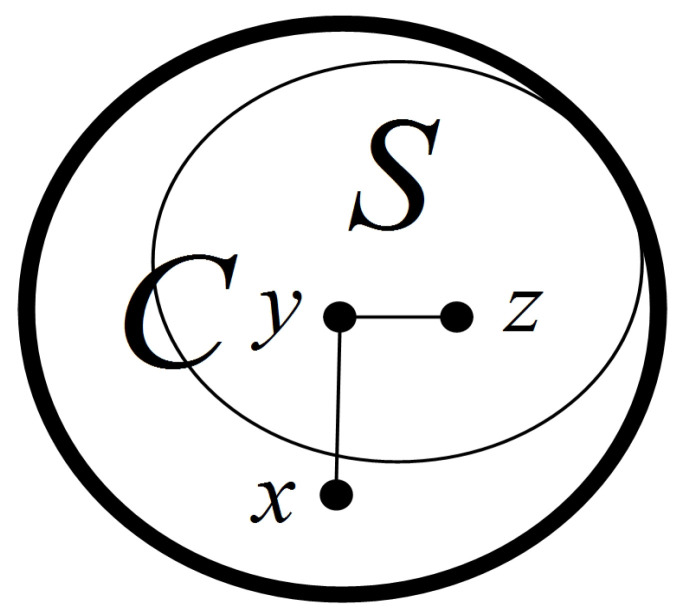
An illustration of Lemma 7.

**Proof** **of** **Lemma** **7.** Since |τ(S)|>|F|, by Lemma 4, all the nodes in *S* are fault-free. Moreover, since *S* is a connected subgraph, *S* belongs to component *C* of G−F, denoted by S⊆C. Suppose that S⊉C. Since *C* is connected, ∃x∈V(C) satisfying x∈N(S) (see Figure 8). We let y∈V(S) such that x∈N(y). Since $S\in T_{0}\left(G\right)$, |V(S)|≥3. There exists another node z∈N(y) with z∈V(S). Furthermore, since *C* is a component of G−F, x,y,z∉F. Thus, $\sigma {\left(x,z\right)}_{y}=0$. By the definition of the 0-test subgraph, x∈V(S), which contradicts x∈N(S). Therefore, S=C.    □

## 4. t/k-Diagnosability and a t/k-Diagnosis Algorithm under the Comparison Model

In the section, we discuss the t/k-diagnosability for a given regular network G=(V,E). The outline of the section is as follows. First, we prove that for a fault set *S* with |S|≤f(k)+1 and k≥0, G−S contains a large component *H* with |V(H)|≥|S| and the number of nodes in G−S−H is no more than k+1 nodes. Next, we discuss the sufficient conditions for the result that *G* is f(k)/k-diagnosable under the comparison model. Finally, based on the obtained sufficient conditions and depth-first search strategy, we design a t/k-diagnosis algorithm for computing a fault set *F* with |F|⩽t for the regular network *G* such that at most *k* free-fault nodes belong to *F*.

Suppose that f(k) is a function of integer *k* with k≥1 and k≤f(k); the following three conditions are used in the rest of this paper.

**Condition** **1.** *For any F⊂V(G) with |F|≤f(k), G−F contains a large component *H* such that |V(H)|≥|V(G)|−|F|−k and |V(H)|≥|F|;*

**Condition** **2.**
|V(G)|≥Δ(G)f(k)+Δ(G)+k+4 *;*

**Condition** **3.** f(k)+1≤f(k+1).

Then, we can derive some theorems and corollaries as follows.

**Corollary** **1.** *Let S be a fault set of G with |S|≤f(k)+1 and k≥0. If Conditions 1 and 3 hold, G−S has a large component H with |V(H)|≥|S|, and the union of the remaining components M has a maximum k+1 nodes.*

**Proof** **of** **Corollary** **1.** Let *F* be a set with |F|≤f(k+1). By Condition 1, G−F contains a large component *L* such that |V(L)|≥|V(G)|−|F|−(k+1) and |V(L)|≥|F|.

By Condition 3, f(k)+1≤f(k+1). According to the conclusion of the previous paragraph, for any S⊂V(G) with |S|≤f(k)+1≤f(k+1), G−S has a large component *H* such that |V(H)|≥|V(G)|−|S|−(k+1) and |V(H)|≥|S|.    □

**Theorem** **1.** *Let F be a fault set of G with |F|≤f(k)+1. If Condition 2 holds and G−F contains a large component L with |V(G)|−|F|−(k+1)⩽|V(L)|, then $L\in T_{0}\left(G\right)$ with |τ(L)|>|F|.*

**Proof** **of** **Theorem** **1.** By Condition 2 and |F|≤f(k)+1, we can obtain

$$\begin{array}{c} \left|V(L)|\geq |V(G)|-|F|-(k+1\right)\\ \geq \Delta (G)f(k)+\Delta (G)+k+4-(f(k)+1)-(k+1)\\ =(\Delta (G)-1)f(k)+\Delta (G)+2. \end{array}$$

Therefore, we have |V(L)|≥3, for Δ(G)≥1.

Since |V(L)|≥3 and *L* is a connected component, Δ(L)≥2. By Lemma 3, we have $|V_{2}\left(L\right)|\geq \frac{|V(L)|-2}{\Delta (L)-1}\geq \frac{\left(\Delta (G)-1)f(k)+\Delta (G\right)}{\Delta (G)-1}>f\left(k\right)+1\geq \left|F\right|$. By Lemma 5, *L* is a 0-test subgraph under the comparison model, denoted by $L\in T_{0}\left(G\right)$. Moreover, by Lemma 6, we have $\left|\tau \left(L\right)|=\right|V_{2}\left(L\right)|>|F|$.    □

**Theorem** **2.** *If Conditions 1 and 2 hold, G is f(k)/k-diagnosable under the comparison model.*

**Proof** **of** **Theorem** **2.** Let *F* be a fault set of *G* with |F|≤f(k). According to Condition 1, G−F contains a large component *L* with |V(L)|≥|V(G)|−|F|−k. By Theorem 1, $L\in T_{0}\left(G\right)$ with |τ(L)|>|F|. That is, there exists a 0-test subgraph *L* such that |V(L)|≥|V(G)|−|F|−k and |τ(L)|>|F|. By Lemma 7, all the nodes in *L* can be identified as fault-free. Since |V(L)|≥|V(G)|−|F|−k, there are fewer than |F|+k nodes that are unidentified. Hence, all the faulty nodes can be isolated into a node set, in which the number of fault-free nodes is no more than *k*. Therefore, under the comparison model, *G* is f(k)/k-diagnosable.    □

Furthermore, we continue to search for a higher value of *t* such that the system is t/k-diagnosable.

**Theorem** **3.** *If Conditions 1–3 hold, then, under the comparison model, G is f(k)+1/k-diagnosable.*

**Proof** **of** **Theorem** **3.** Let *F* be a fault set of *G* with f(k)+1≥|F|. Now, we discuss the situation by considering the following scenarios.

Case 1. |F|≤f(k)

According to Condition 1, G−F contains a large component *H* with |V(H)|≥|V(G)|−|F|−k and |V(H)|≥|F|. By Theorem 1, $H\in T_{0}\left(G\right)$ with |τ(H)|>|F|. Moreover, by Lemma 7, all the nodes in *H* can be identified as fault-free. Since |V(H)|≥|V(G)|−|F|−k, there are fewer than |F|+k unidentified nodes. Therefore, all the faulty nodes can be isolated in a node set containing a maximum bound of *k* fault-free nodes.

Case 2. |F|=f(k)+1

**Figure 9:**
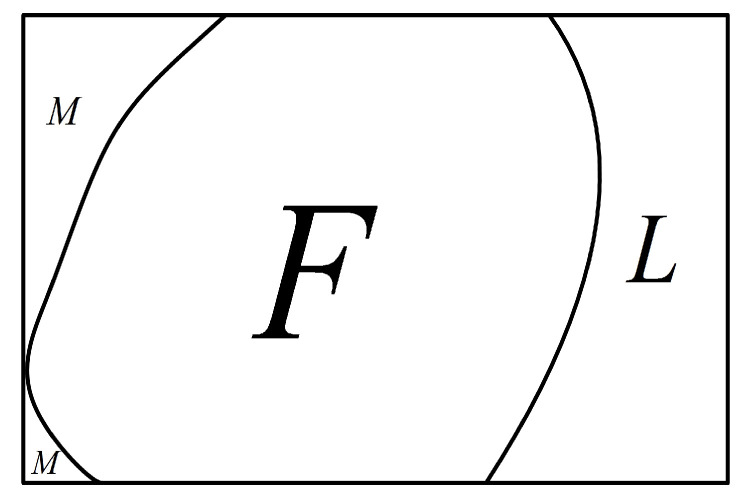
An illustration of case 2.

By Corollary 1, G−F has a large component *L* with |V(L)|≥|F|, and the union of the remaining components *M* has a maximum of k+1 nodes (see Figure 9). We have N(M)⊆F. By Theorem 1, *L* is a 0-test subgraph with |τ(L)|>|F|. Hence, by Lemma 7, all the nodes in *L* can be identified as fault-free. There is a total of |F|+|M| nodes that remain unidentified.

Case 2.1. |M|≤k.

Since |M|≤k, all faulty nodes can be isolated within a node set that at most *k* fault-free nodes are contained.

Case 2.2. |M|=k+1.

**Figure 10:**
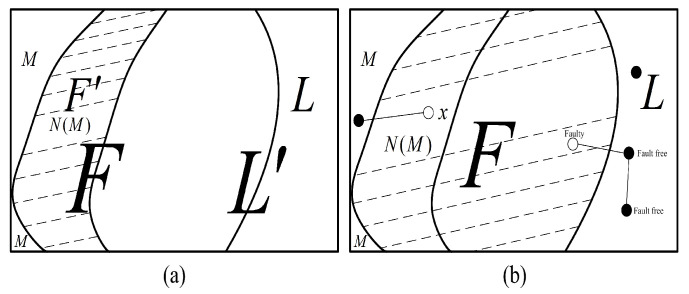
An illustration of case 2.2: (**a**) An illustration of $F^{\prime }=N\left(M\right)$ and $L^{\prime }$. (**b**) An illustration of showing F⊆N(L) and identifying *F* to be fault set.

Suppose that |N(M)|≤f(k). Let $F^{\prime }=N\left(M\right)$; we have $|F^{\prime }|\leq f\left(k\right)$. By Condition 1, $G-F^{\prime }$ has a large component $L^{\prime }$ and a union of remaining components $M^{\prime }$ with $|M^{\prime }|\leq k$ (see Figure 10a). Since N(M)⊆F and $F^{\prime }=N\left(M\right)$, $F^{\prime }\subseteq F$. Therefore, $L\subseteq L^{\prime }$ and $M\subseteq M^{\prime }$. Then, $|M^{\prime }|\geq |M|=k+1$, which contradicts $|M^{\prime }|\leq k$. Therefore, |N(M)|≥f(k)+1. Since |F|=f(k)+1 and N(M)⊆F, we have N(M)=F and |N(M)|=|F|=f(k)+1. That is, each node in *F* has a neighbor in *M*.

Suppose that x∈F and x∉N(L) (see Figure 10b). Let $F^{\prime \prime }=F-\left\{x\right\}$; we have $|F^{\prime \prime }|=f\left(k\right)$. According to Condition 1, $G-F^{\prime \prime }$ has a union of smaller components $M^{\prime \prime }$ with $|M^{\prime \prime }|\leq k$ and a large component. Then, $M^{\prime \prime }=M+\left\{x\right\}$. Therefore, $|M^{\prime \prime }|=|M|+1\geq k+2$, which contradicts $|M^{\prime \prime }|\leq k$. Hence, each node in *F* is connected to at least one neighbor in *L*. That is, F⊆N(L).

Since all the nodes belonging to *L* are fault-free, all the nodes in *F* can be identified as faulty (see Figure 10b), where |F|=f(k)+1. Note that f(k)+1≥|F|, all nodes in *M* are identified as fault-free. Thus, all faulty nodes can be isolated within a node set, and no fault-free node is misidentified as faulty. Therefore, under the comparison model, *G* is f(k)+1/k-diagnosable.    □

Inspired by Lin et al. [8], we introduce a *t*/*k*-diagnosis Algorithm 1 under the comparison model.
**Algorithm 1:** *t*/*k*-diagnosis algorithm under the comparison model**Require**: Conditions 1–3.**Ensure**: $\left(H,F_{i}\right)$, where *H* is the set of nodes that are identified as fault-free and $F_{i}$ is the set of nodes that are isolated.**Step 1**. H=⌀, $F_{i}=⌀$;**Step 2**. Use a depth-first traversal algorithm to derive all the 0-test units under the comparison model;**Step 3**. Obtain the tester of each 0-test units and merge 0-test units to construct $T_{0}\left(G\right)$, and set $T_{0}\left(G\right)=\left\{S_{1},\ldots ,S_{r}\right\}$;**Step 4**. Compute $\left|\tau (S_{i})\right|$ for 1≤i≤r, by merging testers in $S_{i}$;**Step 5**. For each 0-test subgraph $S_{i}$, if $|\tau (S_{i})|>t$, then $H\leftarrow S_{i}$;**Step 6**. $F_{i}\leftarrow N\left(H\right)$;**Step 7**. If $|F_{i}|=t$, then $H\leftarrow V\left(G\right)-H-F_{i}$; else, $F_{i}\leftarrow V\left(G\right)-H-F_{i}$;**Step 8**. Return $\left(H,F_{i}\right)$.

The correctness of the t/k-diagnosis algorithm under the comparison model follows from Theorem 3. In this algorithm, steps 1 and 4–8 take O(1) time. In step 2, the main computational process is based on pairs of adjacent edges. There are $\sum_{x\in V(G)}\deg(x)(\deg(x)-1)/2$ pairs of adjacent edges. Step 3 is based on 0-test units. In the worst case, step 3 need $\sum_{x\in V(G)}\deg(x)(\deg(x)-1)/2$ iterations to compare each pair of 0-test units to see if they have a common test edge. Take an *n*-dimensional hypercube network $Q_{n}$ as an example, $Q_{n}$ is an *n*-regular graph with $|V\left(Q_{n}\right)|=2^{n}$ [11]. Let $N=|V(Q_{n})|$, we have n=logN. Then, $\sum_{x\in V(Q^{n})}\deg(x)(\deg(x)-1)/2=2^{n}\cdot n\left(n-1\right)/2=N\log N\left(\log N-1\right)/2$. Hence, steps 2 and 3 take $O(N\log^{2}N)$ time. As a result, the total time needed by this algorithm for n-dimensional hypercube networks is $O(N\log^{2}N)$, where $N=|V(Q_{n})|$.

## 5. Applications

### 5.1. Applications to Hypercube-like Networks

**Figure 11:**
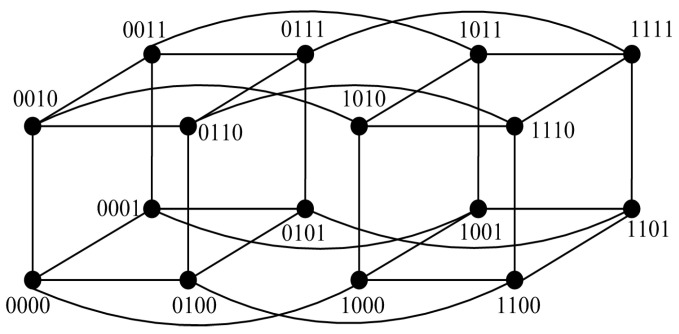
Topology of $H_{n}$ for n=4.

Hypercube-like networks are a class of networks (also called BC networks), which are defined recursively by a perfect matching operation [11] (see Figure 11). An *n*-dimensional hypercube-like network is written as $H_{n}$, where $|V\left(H_{n}\right)|=2^{n}$ [11]. Since $H_{n}$ is *n*-regular [12], we have $\Delta (H_{n})=n$. Note that both n>0 and k>0 are integers, let $f\left(k\right)=n\left(k+1\right)-\frac{k^{2}+3k+2}{2}$; then, we have f(k)≥k for k≤n−1. Then, $H_{n}$ has the following properties.

**Lemma** **8.** *$|V\left(H_{n}\right)|\geq 2f\left(k\right)+k+1$, where n≥4 and k≤n−1.*

**Proof** **of** **Lemma** **8.** Since $|V\left(H_{n}\right)|=2^{n}$ and k≤n−1, we have

$$\begin{array}{c} \left|V\left(H_{n}\right)|-(2f\left(k\right)+k+1\right)\\ =2^{n}-2n(k+1)+k^{2}+3k+2-k-1\\ ={\left(k+(1-n)\right)}^{2}+2^{n}-n^{2}\\ \geq 0,\mathrm{for}n\geq 4. \end{array}$$

Therefore, $|V\left(H_{n}\right)|\geq 2f\left(k\right)+k+1$ for n≥4. □

**Lemma** **9** ([13,14])**.** *Let g and n be two positive integers with g≤n−3 and n≥4, and let $F\subset V(H_{n})$ with $\left|F\right|\leq ng-\frac{\left(g-1)(g+2\right)}{2}-1$. If $H_{n}-F$ is disconnected, there exists a large component in $H_{n}-F$ that includes a minimum of $2^{n}-\left|F\right|-\left(g-1\right)$ nodes.*

**Corollary** **2.** *Let $F\subset V(H_{n})$ with |F|≤f(k), 0≤k≤n−4 and n≥4. If $H_{n}-F$ is disconnected, $H_{n}-F$ has a large component L and a union of smaller components of at most k nodes, where |V(L)|≥|F|.*

**Proof** **of** **Corollary** **2.** Let k=g−1; we have $\left|F\right|\leq f\left(k\right)=f\left(g-1\right)=ng-\frac{{\left(g-1\right)}^{2}+3\left(g-1\right)+2}{2}=ng-\frac{\left(g-1)(g+2\right)}{2}-1$. By Lemma 9, $H_{n}-F$ has a large component *L* and a union of smaller components of at most *k* nodes. By Lemma 8, $|V\left(H_{n}\right)|\geq 2f\left(k\right)+k+1$. Then, we have

$$\begin{array}{c} |V\left(L\right)|\geq |V\left(H_{n}\right)|-|F|-k\\ \geq 2f(k)+k+1-f(k)-k\\ =f(k)+1\\ >|F|. \end{array}$$

□

**Lemma** **10.** *$|V\left(H_{n}\right)|\geq \Delta \left(H_{n}\right)f\left(k\right)+\Delta \left(H_{n}\right)+k+4$, where n≥10 and 0≤k≤n−4.*

**Proof** **of** **Lemma** **10.** Since 0≤k≤n−4 and $\Delta (H_{n})=n$, we have

$$\begin{array}{c} \left|V\left(H_{n}\right)|-(\Delta (H_{n})f\left(k\right)+\Delta (H_{n})+k+4\right)\\ =2^{n}-n^{2}(k+1)+\frac{n(k^{2}+3k+2)}{2}-(n+k+4)\\ \geq 2^{n}-n^{2}(k+1)+n-(n+k+4)\\ =2^{n}-n^{2}(k+1)-(k+4)\\ \geq 2^{n}-n^{2}(n-3)-n\\ =2^{n}-n^{3}+3n^{2}-n\\ >0\mathrm{for}n\geq 10. \end{array}$$

Hence, when n≥10 and 0≤k≤n−4, it holds that $|V\left(H_{n}\right)|\geq \Delta \left(H_{n}\right)f\left(k\right)+\Delta \left(H_{n}\right)+k+4$. □

**Lemma** **11.** *f(k)+1≤f(k+1) for k≤n−3.*

**Proof** **of** **Lemma** **11.** Since $f\left(k\right)=n\left(k+1\right)-\frac{k^{2}+3k+2}{2}$ for k≤n−1, we have

$$\begin{array}{c} f(k)+1-f(k+1)\\ =n\left(k+1\right)-\frac{k^{2}+3k+2}{2}+1-\left(n\left(k+2\right)-\frac{{\left(k+1\right)}^{2}+3\left(k+1\right)+2}{2}\right)\\ =k+3-n\leq 0,\mathrm{for}\;k\leq n-3. \end{array}$$

Therefore, f(k)+1≤f(k+1) for k≤n−2. □

Then, the following result can be derived.

**Theorem** **4.** *$H_{n}$ is f(k)+1/k-diagnosable under the comparison model for 0≤k≤n−4 and n≥10.*

**Proof** **of** **Theorem** **4.** By Corollary 2 and Lemmas 10 and 11, $H_{n}$ satisfies Conditions 1–3 for 0≤k≤n−4 and n≥10. By Theorem 3, it is true that under the comparison model $H_{n}$ is f(k)+1/k-diagnosable. □

### 5.2. Applications to Folded Hypercubes

**Figure 12:**
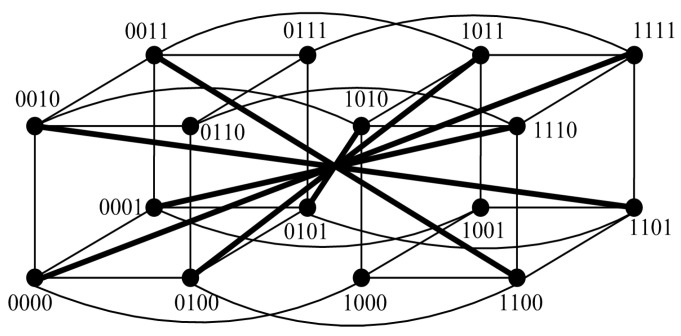
Topology of $FH_{n}$ for n=4.

An *n*-dimensional folded hypercube $FQ_{n}$ is constructed by augmenting a hypercube $Q_{n}$ with 2n−1 extra edges (see Figure 12), where $|V\left(FQ_{n}\right)|=2^{n}$ and $\Delta (FQ_{n})=n+1$ [15]. Let $f\left(k\right)=\left(n+1\right)\left(k+1\right)-\frac{k^{2}+3k+2}{2}$ for k≤n−1; then, the following properties can be derived.

**Lemma** **12.** *$|V\left(FQ_{n}\right)|\geq 2f\left(k\right)+k+1$, where 6⩽n and k≤n−1.*

**Proof** **of** **Lemma** **12.** Since $|V\left(FQ_{n}\right)|=2^{n}$ and k≤n−1, we have

$$\begin{array}{c} |V\left({FQ}_{n}\right)|-\left(2f\left(k\right)+k+1\right)\\ =2^{n}-2\left(n+1\right)\left(k+1\right)+k^{2}+3k+2-k-1\\ ={\left(k-n\right)}^{2}+2^{n}-{\left(n+1\right)}^{2}\\ \geq 0,\mathrm{for}\;n\geq 6. \end{array}$$

Therefore, $|V\left(H_{n}\right)|\geq 2f\left(k\right)+k+1$ for n≥6. □

**Lemma** **13** ([16])**.** *Given two positive integers n and g with n≥6 and $1\leq g\leq \frac{n-1}{2}$, let $F\subset V(FQ_{n})$ with $\left|F\right|\leq \left(n+1\right)g-\frac{1}{2}\left(g^{2}+g\right)$. If $FQ_{n}-F$ is disconnected, $FQ_{n}-F$ has a large component and a union of smaller components of at most g−1 nodes.*

**Corollary** **3.** *Suppose that n≥6 and $0\leq k\leq \frac{n-3}{2}$ are integers, let $F\subset V(FQ_{n})$ with |F|≤f(k). If $FQ_{n}-F$ is disconnected, $FQ_{n}-F$ has a large component L and a union of smaller components of at most k nodes such that |V(L)|≥|F|.*

**Proof** **of** **Corollary** **3.** Let k=g−1, $f\left(k\right)=f\left(g-1\right)=\left(n+1\right)g-\frac{{\left(g-1\right)}^{2}+3\left(g-1\right)+2}{2}=\left(n+1\right)g-\frac{1}{2}\left(g^{2}+g\right)$. By Lemma 13, if $FQ_{n}-F$ is disconnected, $FQ_{n}-F$ has a large component and a union of smaller components of at most *k* nodes. By Lemma 12, $|V\left(FQ_{n}\right)|\geq 2f\left(k\right)+k+1$. Then, we have

$$\begin{array}{c} |V\left(L\right)|\geq |V\left(FQ_{n}\right)|-|F|-k\\ \geq 2f(k)+k+1-f(k)-k\\ =f(k)+1\\ >|F|. \end{array}$$

□

**Lemma** **14.** *$|V\left(FQ_{n}\right)|\geq \Delta \left(FQ_{n}\right)f\left(k\right)+\Delta \left(FQ_{n}\right)+k+4$, where 10⩽n and 0≤k≤n−3.*

**Proof** **of** **Lemma** **14.** Since 0≤k≤n−3 and $\Delta (FQ_{n})=n+1$, we have

$$\begin{array}{c} |V\left(FQ_{n}\right)|-\left(\Delta \left(FQ_{n}\right)f\left(k\right)+\Delta \left(FQ_{n}\right)+k+4\right)\\ =2^{n}-{\left(n+1\right)}^{2}\left(k+1\right)+\frac{\left(n+1\right)\left(k^{2}+3k+2\right)}{2}-\left(n+k+5\right)\\ \geq 2^{n}-\left(n^{2}+2n+1\right)\left(k+1\right)-\left(k+4\right)\\ \geq 2^{n}-\left(n^{2}+2n+1\right)\left(n-2\right)-\left(n+1\right)\\ =2^{n}-n^{3}+2n-1\\ >0,for\;n\geq 10. \end{array}$$

Hence, when 10⩽n and 0≤k≤n−3, $|V\left(FQ_{n}\right)|\geq \Delta \left(FQ_{n}\right)f\left(k\right)+\Delta \left(FQ_{n}\right)+k+4$. □

**Lemma** **15.** *f(k)+1≤f(k+1) for k≤n−2.*

**Proof** **of** **Lemma** **15.** Since $f\left(k\right)=\left(n+1\right)\left(k+1\right)-\frac{k^{2}+3k+2}{2}$ for k≤n−1, we have

$$\begin{array}{c} f(k)+1-f(k+1)\\ =\left(n+1\right)\left(k+1\right)-\frac{k^{2}+3k+2}{2}+1-\left[\left(n+1\right)\left(k+2\right)-\frac{{\left(k+1\right)}^{2}+3\left(k+1\right)+2}{2}\right]\\ =k+2-n\leq 0,\mathrm{for}\;k\leq n-2. \end{array}$$

Therefore, f(k)+1≤f(k+1) for k≤n−2. □

Then, we can obtain the following theorem.

**Theorem** **5.** *$FQ_{n}$ is f(k)+1/k-diagnosable under the comparison model for n≥10 and 0≤k≤n−3.*

**Proof** **of** **Theorem** **5.** By Corollaries 3 and Lemmas 14–15, Conditions 1–3 hold for and 0≤k≤n−3. Therefore, by Theorem 3, $H_{n}$ is f(k)+1/k-diagnosable under the comparison model for n≥10 and 0≤k≤n−3. □

### 5.3. Applications to Star Graphs

**Figure 13:**
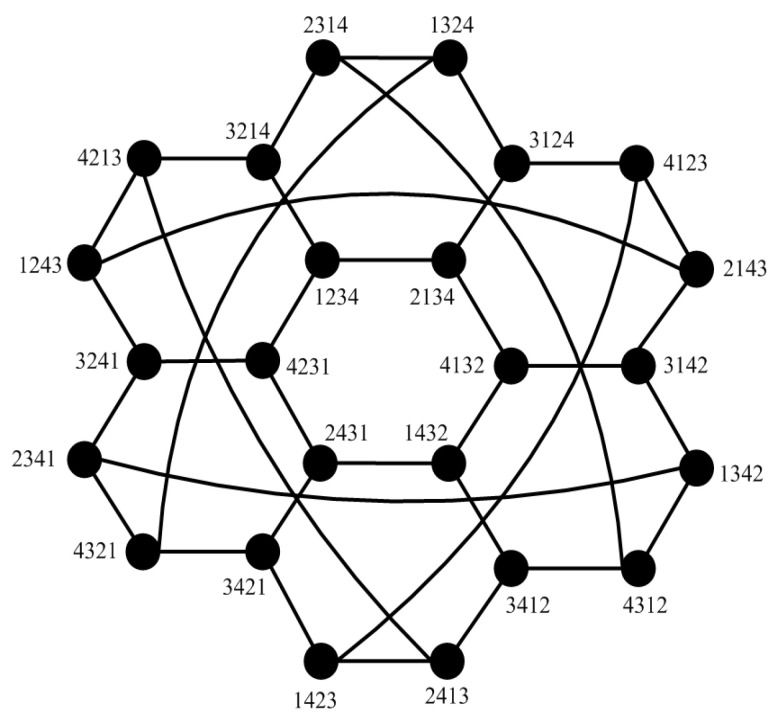
Topology of $S_{n}$ for n=4.

The star graph $S_{n}$ is a sparsely connected graph with $|V(S_{n})|=n!$ and $\Delta (S_{n})=n-1$ [17]. Figure 13 shows $S_{n}$ for n=4. Let f(k)=n(k+1)−3k−2, where k∈{1,2,3}; then, $S_{n}$ has the following lemmas.

**Lemma** **16.** *$|V\left(S_{n}\right)|\geq 2f\left(k\right)+k+1$ for n≥4 and 1≤k≤3.*

**Proof** **of** **Lemma** **16.** Since $|V(S_{n})|=n!$ and n≥4, we have

$$\begin{array}{c} |V\left(S_{n}\right)|-\left(2f\left(k\right)+k+1\right)\\ =n!-2[n(k+1)-3k-2]-k-1\\ =n!-2nk-2n+6k+4-k-1\\ =n!-2nk-2n+5k+3\\ =n!-2n(k+1)+5k+3\\ \geq 0,for\;1\leq k\leq 3. \end{array}$$

Therefore, $|V\left(S_{n}\right)|\geq 2f\left(k\right)+k+1$ for n≥4 and 1≤k≤3. □

**Lemma** **17** ([18,19])**.** *Suppose that n≥4 and F is a subset of $S_{n}$ such that |F|≤2n−5. $S_{n}-F$ has a large component and at most one singleton.*

**Lemma** **18** ([18,19])**.** *Let F be a subset of $S_{n}$ with |F|≤3n−8 and n≥4. $S_{n}-F$ consists of a large component and a collection of smaller components containing no more than two nodes.*

**Lemma** **19** ([17])**.** *Let F be a subset of $S_{n}$ with |F|≤4n−11 and n≥4. If $S_{n}-F$ is disconnected, $S_{n}-F$ has a large component and a union of smaller components of at most three nodes.*

Motivated by Lemmas 16–19, we have the following lemmas.

**Lemma** **20.** *Let F be a subset of $S_{n}$ with |F|≤f(k), n≥4 and 1≤k≤3. If $S_{n}-F$ is disconnected, $S_{n}-F$ consists of a large component L and a collection of smaller components containing no more than k nodes such that |V(L)|≥|F|.*

**Proof** **of** **Lemma** **20.** By Lemmas 17–19, $S_{n}-F$ consists of a large component *L* and a collection of smaller components containing no more than *k* nodes for 1≤k≤3. Then, by Lemma 16, we have

$$\begin{array}{c} |V\left(L\right)|\geq |V\left(S_{n}\right)|-|F|-k\\ \geq 2f(k)+k+1-|F|-k\\ \geq 2f(k)+k+1-f(k)-k\\ =f(k)+1\\ >|F|. \end{array}$$

Hence, |V(L)|≥|F|. □

**Lemma** **21.** *Suppose that 5⩽n and 1≤k≤3. Then, $|V\left(S_{n}\right)|\geq \Delta \left(S_{n}\right)f\left(k\right)+\Delta \left(S_{n}\right)+k+4$.*

**Proof** **of** **Lemma** **21.** We have $\Delta (S_{n})=n-1$ and $|V(S_{n})|=n!$ [17]. Since 1≤k≤3 and n≥5,

$$\begin{array}{l} \left|V\left(S_{n}\right)\right|-\left(\Delta \left(S_{n}\right)f\left(k\right)+\Delta \left(S_{n}\right)+k+4\right)\\ =n!-n\left(n-1\right)\left(k+1\right)+\left(n-1\right)\left(3k+2\right)-\left(n+k+3\right)\\ =n!-\left(k+1\right)n^{2}+\left(3k+1\right)n-\left(3k+4\right)\\ >n!-\left(k+1\right)n^{2}\\ \geq n!-4n^{2}\\ >0. \end{array}$$

Hence, when 5⩽n and 1≤k≤3, it is true that $|V\left(S_{n}\right)|\geq \Delta \left(S_{n}\right)f\left(k\right)+\Delta \left(S_{n}\right)+k+4$. □

**Lemma** **22.** *f(k)+1≤f(k+1) for 1≤k≤3 and n≥4.*

**Proof** **of** **Lemma** **22.** Note that f(k)=n(k+1)−3k−2, where 1≤k≤3, we have

$$\begin{array}{c} f(k)+1-f(k+1)\\ =n\left(k+1\right)-3k-2+1-[n\left(k+2\right)-3(k+1)-2]\\ =-n+4\\ \geq 0,for\;n\geq 4. \end{array}$$

Therefore, f(k)+1≤f(k+1) for 1≤k≤3 and n≥4. □

Therefore, for f(k)=n(k+1)−3k−2, we obtain the following theorem.

**Theorem** **6.** *$S_{n}$ is f(k)+1/k-diagnosable under the comparison model for n≥5 and 1≤k≤3.*

**Proof** **of** **Theorem** **6.** By Lemmas 20–22, Conditions 1–3 hold for n≥5 and 1≤k≤3. Therefore, by Theorem 3, $S_{n}$ is f(k)+1/k-diagnosable under the comparison model for n≥5 and 1≤k≤3. □

### 5.4. Applications to Complete Cubic Networks

**Figure 14:**
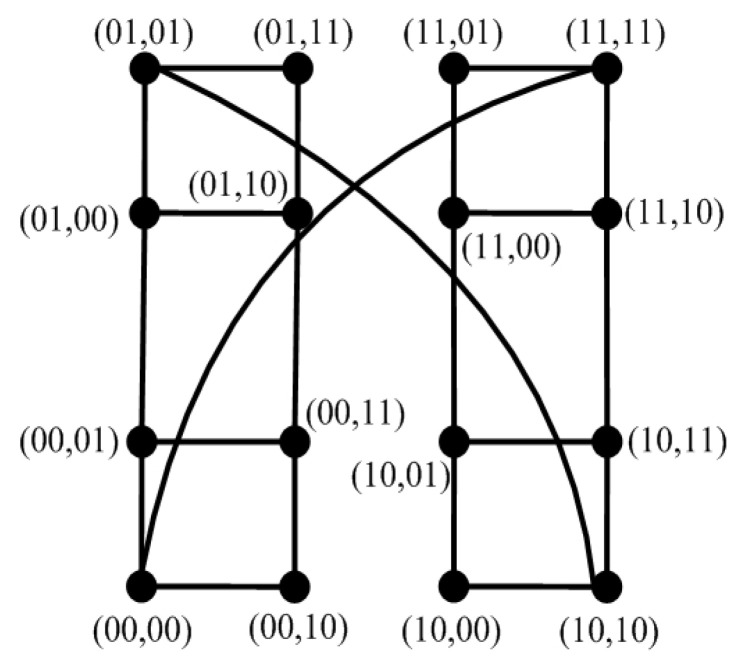
The topology of CN(n) for n=2.

An n-dimensional complete cubic network, written as CN(n), is a special class of hierarchical cubic networks [20]. Figure 14 shows CN(n) for n=2. According to the definition of CN(n), we have $\left|V\left(CN\left(n\right)\right)\right|=2^{2n}$ and Δ(CN(n))=n+1. Let $f\left(k\right)=n\left(k+1\right)-\frac{k(k+1)}{2}$ for 0≤k≤n−1; then, the following properties can be obtained.

**Lemma** **23.** *|V(CN(n))|≥2f(k)+k+1 for n≥2 and 0≤k≤n−1.*

**Proof** **of** **Lemma** **23.** Since $\left|V\left(CN\left(n\right)\right)\right|=2^{2n}$ and 0≤k≤n−1, we have

$$\begin{array}{c} \left|V\left(CN\left(n\right)\right)\right|-\left(2f(k)+k+1\right)\\ =2^{2n}-2(n(k+1)-\frac{k(k+1)}{2})-k-1\\ =2^{2n}-2nk-2n+k^{2}+k-k-1\\ =2^{2n}-2nk-2n+k^{2}-1\\ =2^{2n}-2(k+1)n+k^{2}-1\\ =2^{2n}-2n^{2}\\ \geq 0,for\;n\geq 2. \end{array}$$

Therefore, |V(CN(n))|≥2f(k)+k+1 for n≥2 and 0≤k≤n−1. □

**Lemma** **24** ([20])**.** *Let n≥2 and 1≤g≤n. For any F⊂V(CN(n)) with $\left|F\right|\leq gn-\frac{g(g-1)}{2}$, there exists a large component and several smaller components containing a maximum of g−1 nodes in CN(n)−F.*

By Lemma 24, we can deduce the following corollary.

**Corollary** **4.** *Let F⊂V(CN(n)) with n≥2, 0≤k≤n−1 and $\left|F\right|\leq n\left(k+1\right)-\frac{k(k+1)}{2}$. In CN(n)−F, there exists a large component L and several smaller components containing a maximum of k nodes, where |V(L)|≥|F|.*

**Proof** **of** **Corollary** **4.** By Lemma 24, let k=g−1, we have $\left|F\right|\leq ng-\frac{g(g-1)}{2}$. Then, there exists a large component *L* and several smaller components containing a maximum of *g* nodes in CN(n)−F, where n≥2 and 0≤k≤n−1. Then, we have |V(L)|≥|V(CN(n))|−|F|−k. By Lemma 23,

$$\begin{array}{c} |V(L)|\geq |V(CN(n))|-|F|-k\\ \geq 2f(k)+k+1-|F|-k\\ \geq 2f(k)+k+1-f(k)-k\\ =f(k)+1\\ >|F|. \end{array}$$

Hence, |V(L)|≥|F|. □

**Lemma** **25.**
|V(CN(n))|≥Δ(CN(n))f(k)

*+Δ(CN(n))+k+4 for 0≤k≤n−1 and n≥3.*

**Proof** **of** **Lemma** **25.** We have $\left|V\left(CN\left(n\right)\right)\right|=2^{2n}$ and Δ(CN(n))=n+1. Since 0≤k≤n−1 and n≥3,

$$\begin{array}{c} \left|V\left(CN\left(n\right)\right)\right|-(\Delta (CN(n))f\left(k\right)+\Delta (CN(n))+k+4)\\ =2^{2n}-n(n+1)(k+1)+\frac{\left(n+1)(k^{2}+k\right)}{2}-(n+k+5)\\ \geq 2^{2n}-\left(n^{2}+n)(k+1)-(n+k+5\right)\\ \geq 2^{2n}-\left(n^{3}+n^{2}+2n+4\right)\\ >0. \end{array}$$

Hence, |V(CN(n))|≥Δ(CN(n))f(k)+Δ(CN(n))+k+4 for 0≤k≤n−1 and n≥3. □

**Lemma** **26.** *f(k)+1≤f(k+1) for 0≤k≤n−2.*

**Proof** **of** **Lemma** **26.** Since $f\left(k\right)=n\left(k+1\right)-\frac{k(k+1)}{2}$ for 0≤k≤n−2, we have

$$\begin{array}{l} f\left(k\right)+1-f\left(k+1\right)\\ =n\left(k+1\right)-\frac{k(k+1)}{2}+1-\left[n\left(k+2\right)-\frac{\left(k+1)(k+2\right)}{2}\right]\\ =-n+k+2\\ \leq 0. \end{array}$$

Therefore, f(k)+1≤f(k+1) for 0≤k≤n−2. □

**Theorem** **7.** *CN(n) is f(k)+1/k-diagnosable under the comparison model for n≥3 and 0≤k≤n−2.*

**Proof** **of** **Theorem** **7.** By Corollary 4 and Lemmas 25 and 26, Conditions 1–3 hold for 0≤k≤n−2 and n≥3. Therefore, by Theorem 3, $H_{n}$ is f(k)+1/k-diagnosable for n≥10 and 0≤k≤n−2. □

## 6. Conclusions

t/k-diagnosability is an important diagnostic strategy that can improve the self-diagnosing capability of multiprocessor systems. While significant progress has been made in t/k-diagnosability under the PMC model in the last half century, t/k-diagnosability and t/k-diagnosis algorithms for many regular networks under the comparison model have yet to be determined. In this paper, inspired by the 0-test subgraph under the PMC model, we introduce some useful notions for the comparison model, such as the 0-test unit, 0-test set, and 0-test subgraph. Then, we study the properties of 0-test subgraphs under the comparison model. Furthermore, we derive some key theorems about t/k-diagnosability and the t/k-diagnosis algorithm under the comparison model. Finally, the applications of our results to some regular networks are demonstrated.

In the article, we calculate the t/k-diagnosability for regular networks based on the comparison model. Considering that *N*-ary *M*-cube networks are more general than regular networks in terms of network topology, in the future, we will investigate the t/k-diagnosability problem of *N*-ary *M*-cube networks under the comparison model.

## Figures and Tables

**Table 1:** Invalidation rules for the comparison model.

Comparator *z*	Tested Nodes *x* and *y*	$\sigma {\left(x,y\right)}_{z}$
Fault-free	Fault-free	0
Fault-free	At least one is faulty	1
Faulty	Any case	0 or 1

## Data Availability

Data are contained within the article.
